# Limitations in Determining Oxidation States in Condensed Matter at the Subnanometric Scale

**DOI:** 10.1021/jacs.5c02242

**Published:** 2025-06-13

**Authors:** Deborah Perco, Monica Pozzo, Andrea Berti, Federico Loi, Paolo Lacovig, Silvano Lizzit, Aras Kartouzian, Ueli Heiz, Dario Alfè, Alessandro Baraldi

**Affiliations:** † Department of Physics, 9315 University of Trieste, via Valerio 2, 34127 Trieste, Italy; ‡ Faculty of Technological & Innovation Sciences, Universitas Mercatorum, Piazza Mattei 10, 00186 Rome, Italy; § Institute for Materials Discovery, UCL East, Marshgate Building, 7 Sidings Street, Stratford, London E20 2AE, U.K.; ∥ J. Heyrovsky Institute of Physical Chemistry, Dolejškova 2155/73, 182 23 Prague, Czech Republic; ⊥ Elettra Sincrotrone Trieste, AREA Science Park, 34149 Trieste, Italy; # Chemistry Department & Catalysis Research Center, Technical University of Munich, Lichtenbergstr. 4, Garching D-85748, Germany; ∇ Department of Earth Sciences and London Centre for Nanotechnology, 4919 University College London, Gower Street, London WC1E 6BT, U.K.; ○ Dipartimento di Fisica Ettore Pancini, Università di Napoli Federico II, Monte S. Angelo, 80126 Napoli, Italy

## Abstract

The oxidation state is a fundamental chemical concept commonly employed to rationalize, classify, and predict the chemical reactivity of a variety of compounds. Understanding and defining the elemental oxidation state of solid materials at the atomic level becomes increasingly complex as their physical dimensions are reduced from tens of nanometers—where properties are still dominated by bulk or outer atomic crystal plane characteristics, to the subnanometric limit. In this work, we highlight the significant limitations in determining even a basic quantity, such as the oxidation state, when oxidized low-nuclearity mass-selected clusters are investigated by means of X-ray photoelectron spectroscopy (XPS), widely recognized as the elective approach to resolve different oxidations states. The lack of crystalline order in these nanoclusters, unlike that in periodic bulk systems and in solid surfaces, leads to a broad distribution of measured core levels, as shown in the case study of W nanoclusters. These cannot be unambiguously assigned to a valence state based solely on the knowledge of bulk matter behavior but need close comparison with specific theoretical modeling. Our results emphasize the substantial challenges inherent in understanding the unique properties of nanoscale materials, particularly in making a rigorous and quantitative determination of a fundamental property that takes a relevant role in many chemical processes, and represent crucial knowledge for advancing technologies that rely on the miniaturization of matter in various processes.

## Graphical abstract

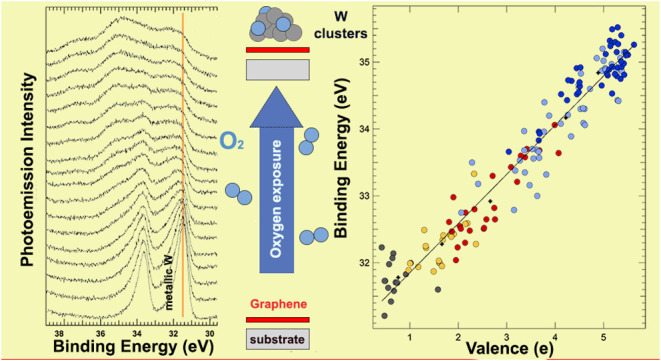

## Introduction

The concept of oxidation state, first introduced by Antoine Lavoisier in the 18th century,^1^ remains a fundamental principle across numerous scientific disciplines today. For example, the abundance of carbon on earth is intricately linked to the oxidation state of iron under the extreme pressures of the earth’s upper mantle,^2^ where specific oxidation conditions significantly influence seismic wave attenuation.^3,4^ The oxidation state of iron also plays a pivotal role in the development of the interstellar medium.^5^ In medicine, Pt^4+^ complexes have been found to be more effective as anticancer agents compared to Pt^2+^ complexes,^6^ while copper’s redox properties are essential for a variety of biological processes.^7^ It takes a fundamental role, especially in material science where the oxidation state of an element is most critical as it governs its chemical, physical, and thermodynamical properties and helps to predict the nature of chemical bonding in new materials. Oxidation processes are key to forming protective layers on copper surfaces, which are vital for applications in the semiconductor industry and electro-optics^8,9^ as well as for controlling nanoscale electrostatic heterojunctions in van der Waals materials.^10^ Moreover, the oxidation state of Pd strongly influences the performance and selectivity of Pd surfaces for hydrogen peroxide synthesis,^11^ while the presence of Ce^3+^ or Ce^4+^ in nonstoichiometric CeO_2_ strongly affects the surface reactivity.^12,13^ To determine the oxidation state of inorganic materials, together with their crystal structure, bond valence (or bond order) theory, as developed by Pauling,^14,15^ is frequently employed. This theory establishes a relationship between the bond length and valence, wherein the sum of bond valences around a particular atom corresponds to its oxidation state. However, even in recent decades, the concept of oxidation state has been the subject of debate and revisions,^16,17^ not only in solid-state systems^18^ but also in liquids^19^ and metal–organic frameworks.^20^ These discussions have been paralleled by critical assessments within the scientific community, culminating in the establishment of a new definition by IUPAC^21^ and alternative frameworks.^22^ From an experimental standpoint, it is widely accepted that core-level photoelectron spectroscopy—commonly referred to as X-ray photoelectron spectroscopy (XPS) or electron spectroscopy for chemical analysis (ESCA)—provides definitive fingerprints for unambiguously determining atomic oxidation states.^23,24^ Since its early development in the 20th century and its widespread adoption through the pioneering work of Siegbahn,^25^ XPS has become an essential analytical tool in various fields of materials science. Its elemental and surface sensitivities make it especially suited for determining oxidation states in both bulk and surface environments, particularly in systems involving strong interionic charge transfer processes.

In this study, we explore whether the photoelectron spectroscopy approach can be successfully applied at the subnanometric scale, where atomic aggregates consist of only a few atoms. The quantum properties at this scale are of critical importance for technological applications, such as heterogeneous catalysis, with small clusters that exhibit enhanced chemical reactivity and selectivity^26–30^ and magnetic data storage, where atomic aggregates show increased magnetic moments compared to their bulk counterparts.^31,32^ To address the challenge of defining and determining the oxidation state at the atomic level, we conducted high-resolution XPS experiments on size-selected tungsten clusters composed of only a few atoms. Tungsten was chosen due to its relevance in fields such as catalysis, electrochemistry, and phototherapy, both in bulk and nanostructured forms.^33–37^ More importantly, tungsten offers a broad range of oxidation states for investigation. These states span from W^2+^, often observed on surfaces^38^ or in polycrystalline tungsten, to W^6+^ in tungsten trioxide (WO_3_),^39^ which is the most studied electrochromic material.^40,41^ Intermediate oxidation states, such as W^4+^ in tungsten dioxide (WO_2_),^42^ are also frequently observed. The W^3+^ state was first reported in 2006 in thin film oxides;^43^ furthermore, tungsten can also form substoichiometric compounds like W_25_O_73_, where W^5+^ is present.^44^ In XPS, the W 4f_7/2_ core-level signals at 31.4, 32.0, 32.5, 34.7, and 36.1 eV serve as fingerprints for metallic tungsten and its 0, 2+, 4+, 5+, and 6+ oxidation states, respectively.^45–48^ Even on solid surfaces, the dissociative adsorption of O_2_ on the close-packed W(110) surface allows for distinct W 4f_7/2_ components corresponding to the formation of single, double, and triple W–O bonds, all positioned at the same 3-fold site.^49,50^ As the number of W–O bonds increases, the core-level binding energies shift almost proportionally.^51^ This behavior is consistent with oxygen interactions on other transition metal surfaces, such as Rh and Ru.^52,53^ Moving to nanostructures, several studies, both theoretical and experimental, have explored the oxidation of tungsten nanoclusters or tungsten oxide nanoclusters, especially at smaller sizes. For example, the geometric and electronic structures of gas-phase (WO_3_)*_n_* (*n* = 1–4) clusters have been examined using density functional theory (DFT) and molecular dynamics (MD) simulations,^54,55^ with (WO_3_)_4_ exhibiting a bulk-like structure. The interaction with the substrate can have a strong influence and lead to a structural distortion, stretching W–O bond lengths in the nanoclusters.^56^ For TiO_2_-supported clusters, W 4f core-level spectra reveal three distinct components: a metallic species at 30.9 eV, shifted by −0.5 eV from bulk tungsten, and components at 34.6 and 35.9 eV, corresponding to W^5+^ and W^6+^, respectively. However, none of these studies explored the evolution of the oxidation of tungsten nanoclusters and the influence of the O atoms on the core levels of tungsten atoms. In this article, we directly address the interdependence of charge, atomic coordination, and bond lengths in assigning oxidation states to tungsten nanoclusters.

## Results and Discussion

Our investigation is focused on the in situ oxidation of tungsten at the subnanometer scale, facilitated by the production and deposition of W-size-selected clusters consisting of 13 and 25 atoms onto graphene. Graphene was epitaxially grown on Ru(0001)^57^ and selected due to the pronounced corrugation of the honeycomb carbon layer, which significantly contributes to limit atomic mobility especially at the low temperature at which we performed our experiments, i.e., 40 K (see Experimental Methods section). Previous DFT calculations demonstrated that the diffusion barrier of a W monomer in the valley region of the moiré pattern is equal to 1.07 eV,^58^ a value that agrees with our computation, i.e., 1.17 eV for the FCC region and 1.06 eV for the HCP region. Given these barriers, the clusters are expected to remain immobile at 40 K, preventing sintering and ensuring the study of clusters with precise nuclearity. Thirteen and 25 sizes were selected because the former is a *magic number* while the latter is the tungsten stoichiometry in one of the substoichiometric phases of tungsten oxides.^59^ Mass selectivity of the clusters was confirmed via mass spectrometry, as shown in Figure 1S of the Supporting Information. To avoid cluster fragmentation during deposition we employed the soft-landing mode.^60^ The cluster density was lower than the 9 × 10^–3^ cluster per nm^2^, to further guarantee mass selection. After the deposition, nanoclusters were exposed to increasing pressures of molecular oxygen (1 × 10^–10^–1 × 10^–9^ mbar), using the photodissociation of oxygen molecules at 40 K, a method proven effective in prior studies with Ag,^61^ Fe,^62^ and Pt^63^ nanoclusters. However, it is important to underline that spontaneous oxygen dissociation occurs on W surfaces even at very low temperatures.^64,65^

To assess the oxidation of these sparse W nanoclusters, we took advantage of the high-photon flux at the SuperESCA beamline of Elettra (see Experimental Methods section), enabling real-time monitoring of W 4f core-level spectra during oxygen exposure, up to a total dose of 1.35 L (1 L = 1 Torr × 1 s). The results for W_13_ and W_25_ clusters are presented in Figure 1 and in Supporting Videos 1 and 2. At first glance, the spectral evolution of the 4f spin–orbit split components (Δ*E* = 2.15 eV) shows marked differences between the two clusters. In both cases, the spectral weight shifts toward higher binding energies with increasing the level of exposure to the O_2_ exposure. However, two key distinctions emerge: (i) W_13_ shows a more pronounced increase in higher binding energy components, and (ii) the signal at 31.45 eV, indicative of metallic W, persists in W_25_ even after extended oxygen exposure. After 1.3 L exposure, the spectral line shape stabilizes, indicating saturation in oxygen adsorption. The complexity of the spectral components, characterized by spin–orbit doublets and, most importantly, by considerable spectral broadening, poses a significant challenge for data analysis compared to simpler systems like atomic or molecular species adsorbed on solid surfaces. To tackle this complexity, we opted to guide our analysis using DFT calculations. For this purpose, we first evaluated the adsorption structure of W_13_ and W_25_ clusters on a graphene layer with a periodicity of (13 × 13) on a (12 × 12) Ru(0001) surface (see Theoretical Methods section). It is well-known that different isomers may be produced during the laser ablation and mass selection process. However, a systematic and extensive statistical evaluation of the core-level states (including final-state effects) of tungsten atoms in systems composed of nearly 1000 atoms is computationally very demanding. For this reason, for each mass, we considered the two gas-phase minimum-energy structures retrieved from the Quantum Cluster Database.^66^ For these, we removed one electron to replicate the ionic species generated in the experiment, relaxed the system, and determined the lowest-energy configuration. These electrically neutralized clusters were placed in the so-called “valleys” of graphene, where the Ru-carbon distance (approximately 2.18 Å) is the shortest.^67^ These configurations were placed in four different rotational orientations on the graphene surface, and the most stable configuration was selected for the study of the core levels under increasing oxygen densities. Although this strategy does not explicitly account for different isomeric conformations, our overall analysis includes a total of 215 nonequivalent W atoms, providing a representative statistical sampling of atomic environments across different isomers. The adsorption in the “valley” FCC site of the moiré unit cell (see Figure 2a) is consistent with observations from other atoms and clusters on the same substrate.^68^ The metallic cluster morphologies appear only slightly distorted upon adsorption on graphene, as shown in Figure 2b, indicating minimal interaction between tungsten and carbon, almost preserving their gas-phase structure.

**Figure 1:**
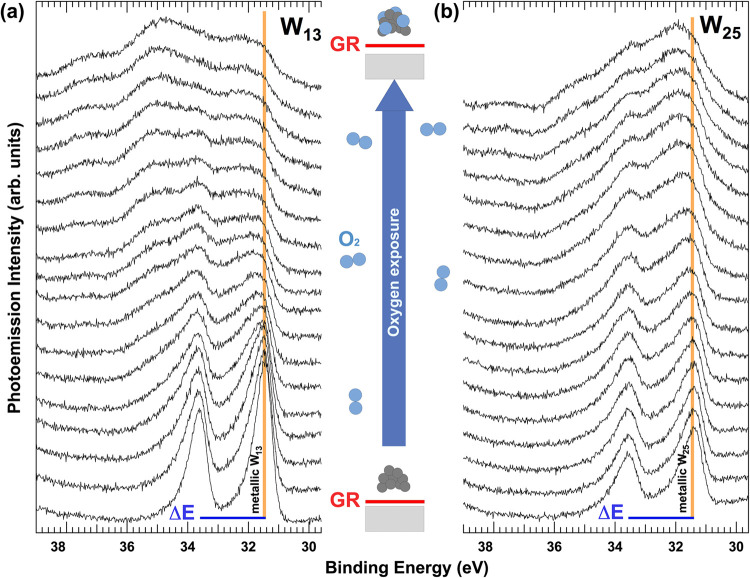
Selected (one every three) W 4f core-level spectral sequence acquired during oxygen exposure at 40 K of the (a) W_13_ and (b) W_25_ nanocluster deposited on the graphene/Ru(0001) interface up to a total dose of 1.35 L (1 L = 1 Torr × 1 s). For clarity, spectra in panel (b) have been normalized in such a way to present comparable intensity. Colored bars indicate the binding energy of the metallic 4f_7/2_ components. Δ*E* is the spin–orbit splitting (Δ*E* = 2.15 eV). Data are reported in the graph between 38.6 and 29.8 eV, while the original data have been acquired in a wider binding energy range to properly consider the line shape of the background (see Experimental Methods).

**Figure 2:**
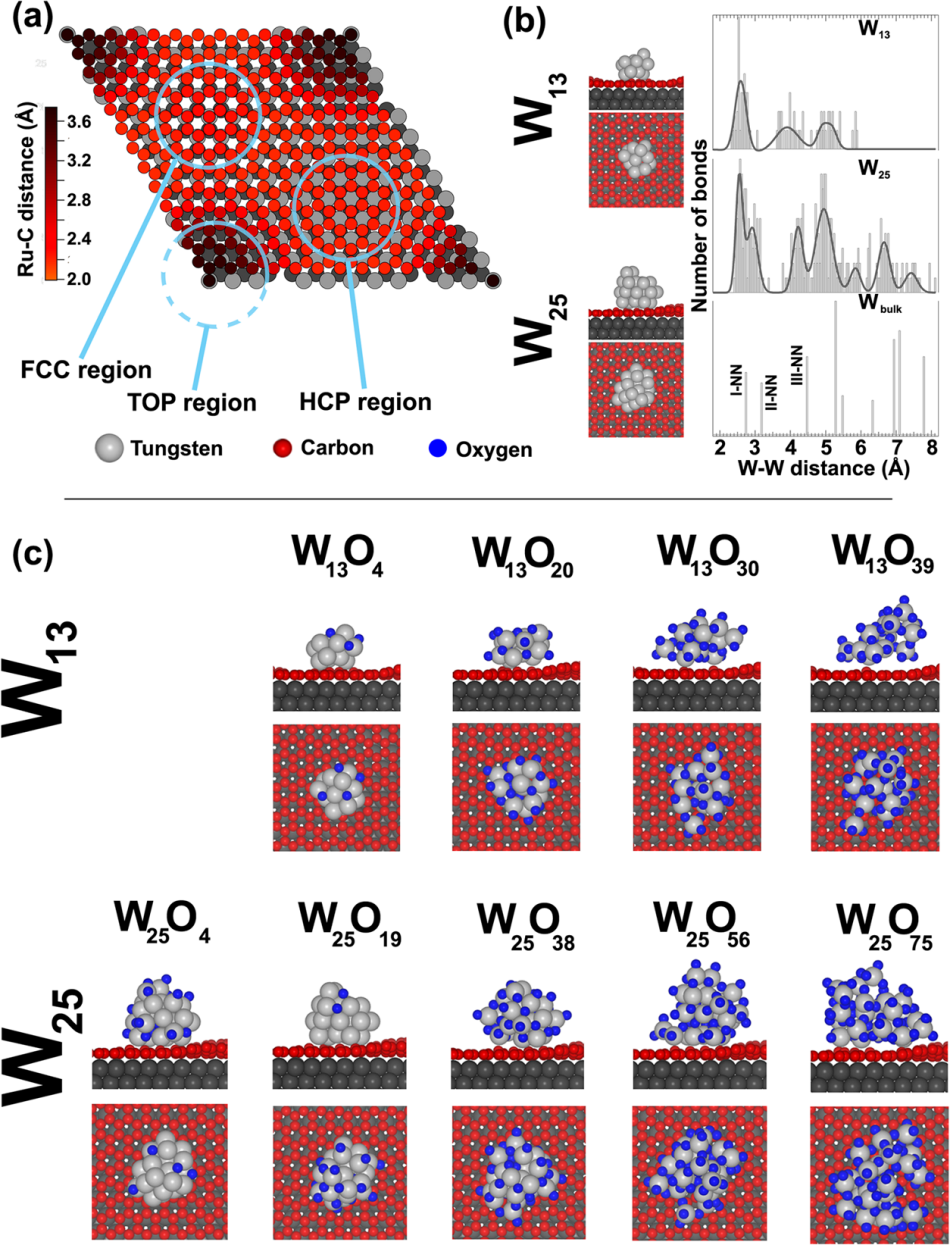
(a) Top view of (13 × 13) carbon – (12 × 12) ruthenium cell. The color scale is used to represent different C–Ru distances. The three different regions of the cell, defined with respect to the matching with the substrate, are also indicated. (b) Top and side views of the DFT calculated relaxed structures of clean W_13_ and W_25_ clusters. The right panel shows the broadened distribution in the W–W distance for both clusters when compared with the W–W distances in bulk tungsten. (c) Top and side views of the DFT calculated relaxed structures of W_13_ (top) and W_25_ (bottom) clusters oxidized with different oxygen densities.

The theoretical structural calculations reveal a notable variability in the W–W interatomic distances within the smaller W_13_ clusters, as illustrated in Figure 2b (top panel), which displays all W–W interatomic distances present in the cluster. While the W–W distances distribution for W_25_ can be fitted with the same peaks found for bulk tungsten, with a small rigid shift toward lower values, W_13_ shows a broader distribution that does not allow to define properly first- (I-NN), second- (II-NN) and third-nearest (III-NN) neighbors. By contrast, for W_25_ (middle panel Figure 2b), typical interatomic distances of bulk tungsten among neighbors can be identified, although there remains a pronounced spread of values. We then systematically introduced increasing amounts of oxygen atoms into the clusters, as depicted in Figure 2c, achieving a maximum W/O ratio close to 1:3. The strategy employed for incorporating oxygen into the clusters before global structural DFT relaxation is described in the Theoretical Methods section. Clusters with four different oxygen densities for W_13_ and five for W_25_ were generated and relaxed, resulting in W_13_O*_m_* (*m* = 4, 20, 30, 39) and W_25_O*_m_* (*m* = 4, 19, 38, 56, 75) as illustrated in Figure 2b.

Oxygen adsorption induces a clear geometric distortion in the clusters, notably increasing the average W–W interatomic distances from 3.74 Å (W_13_) to 5.79 Å (W_13_O_39_) and from 4.62 Å (W_25_) to 7.28 Å (W_25_O_75_) (see Figure 2S in the Supporting Information). Despite these changes, both clusters maintain a three-dimensional structure without exhibiting any tendency to flatten on graphene. This behavior is expected as the strong W–W interactions and the substantial energy barriers associated with structural distortions render such a flattening process highly improbable.

For all configurations presented in Figure 2, W 4f core levels for each tungsten atom in the clusters were calculated using DFT, incorporating final-state effects.^69^ Figure 3a illustrates the calculated core-level shift values for each atom as a function of the average W–O distance. The different W atoms were categorized into six families, each one defined by the same number *n* of bonds between tungsten and oxygen atoms. We identified an O atom as bonded to a W atom if the W–O bond length was less than or equal to 2.2 Å. As anticipated, an increase in *n* (*n* = 1–6) correlates with progressively higher core-level values. This is evident from the core-level trend plotted when varying the Bader charge calculated for all the atoms of the two clusters in the different configurations we probed, as reported in Figure 3S of the Supporting Information. The trend is almost linear, with a linear correlation coefficient of 0.97.

**Figure 3:**
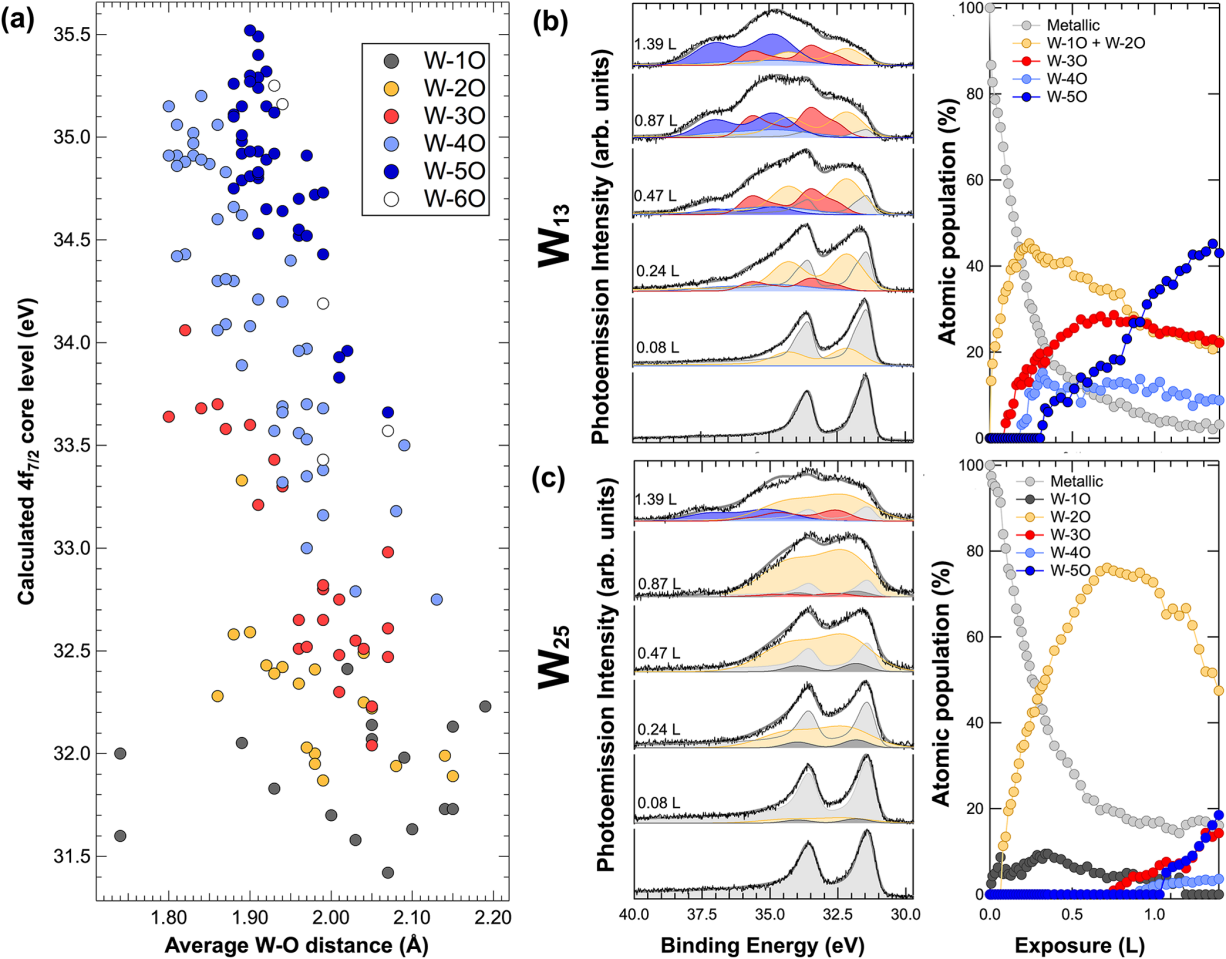
(a) Calculated W 4f_7/2_ core levels as a function of W–O interatomic bonds for W_13_ and for W_25_. Different colors correspond to different families of W atoms characterized by a variable number n of bonds formed by W atoms with oxygen, ranging from 1 to 6. (b) W_13_ core levels spectra fitted using W-*n*O families. (c) W_25_ core levels spectra fitted using W-*n*O families.

Based on the theoretical data regarding the W 4f_7/2_ binding energies of individual tungsten atoms, we constructed the spectral line shape for each of the six W-*n*O families (see Experimental Methods). The results of the fitting procedure for selected spectra (out of a total of 48 for each uptake sequence) corresponding to metallic and oxidized tungsten nanoclusters are depicted in Figure 3b for W_13_ and Figure 3c for W_25_ clusters. The different spectral components are presented in various colors in the left panels, while the evolution of the spectral intensities as a function of oxygen exposure is shown in the right panels. A remarkable finding is the substantial difference in the behavior of the intensity of the metallic component (dark gray peak) for the two clusters at the end of the uptake process. For W_13_, the metallic component vanishes completely by the end of the oxygen exposure, whereas W_25_ retains about 20% of its atoms that remain unbonded to oxygen, which almost matches the percentage of atoms in the cluster core. This observation underscores the significant role played by cluster size in the oxidation process, as outer atomic layers inhibit oxygen penetration toward the cluster’s core at the low temperatures employed in our experiments. As expected, W_25_ atoms, like those of W_13_, form an increasing number of bonds with the adsorbate as oxygen exposure increases. The effect of varying cluster sizes is further reflected in the pronounced growth of the higher binding energy components for W_13_, particularly among the atoms coordinated with five oxygens (W-5O). Interestingly, even at high oxygen densities (W/O = 1:3), the number of W atoms bonded to six oxygens, a configuration typically associated with the ordered crystal structure of bulk tungsten oxides, results in being extremely low. From DFT calculations, we determined that W_13_O_20_ features W atoms coordinated with 2, 3, 4, and 5 O atoms, while W_13_O_30_ includes W atoms coordinated exclusively with 3, 4, or 5 O atoms. This suggests that in our experiments, at the end of the oxygen exposure, the number of atoms adsorbed on W_13_ ranges between 20 and 30. Applying similar reasoning to W_25_ indicates that the number of atoms adsorbed on this cluster falls between 19 and 38. Additionally, for statistical reasons, cluster configurations with slightly different oxygen densities may coexist, complicating the attainment of a consistent stoichiometry for each cluster.

The data analysis, using this approach, however, does not allow for the unambiguous assignment of oxidation states to specific spectral components if we state that the oxidation state and coordination are proportional. This limitation shows up because we found identical core-level binding energies for atoms with different atomic coordination with oxygen, as illustrated in Figure 4S of Supporting Information. This effect is especially prominent at high oxygen concentrations, where tungsten atoms may form 4 or 5 bonds with oxygen. Consequently, a different approach is needed to identify the W oxidation states in W_13_ and W_25_. Indeed, beside the average coordination that we considered, atomic properties are strictly dependent also on valence states, as extensively discussed by Brown.^70^ In bulk tungsten compounds, the coordination with oxygen atoms is equal to 6 for WO_3_ and WO_2_, but the structure of the latter is distorted and shows longer W–O bond lengths with respect to the one of WO_3_. Thus, to better understand the relationship between core levels and oxidation states of matter at the nanoscale, we adopted the formalism developed by Pauling, which correlates the bond valence *S*_*i*_ of an atom *i* to the interatomic bond length (*R*_*ij*_) through the equation *S*_*i*_ = ∑*_j_* *s*_*ij*_ = ∑*_j_* exp­[(*R*_0_ – *R*_*ij*_)/*b*], where *R*_0_ and *b* are constant parameters specific to the atoms involved in the bond, *R*_*ij*_ is the bond length between atoms *i* and *j*, and *s*_*ij*_ are the bond orders between atoms *i* and *j*. According to the bond valence conservation principle, which expresses local neutrality, the sum of the bond orders around a specific atom equals its valence. Following this well-established approach, we computed the valences of all W atoms in the nanoclusters across various oxygen densities. The values of *R*_0_ (1.9 Å) and b (0.33) are standard for W–O bonds in tungsten compounds.^71^ The calculated core levels of each atom plotted as a function of each valence are presented in Figure 4. We did not consider the 6O family in this analysis since it does not appear in the nanocluster’s spectra and since the number of points is small and may lead to wrong conclusions. The graph reveals a strong linear correlation between core levels and valence (Pearson correlation coefficient equal to 0.97), with computed mean values of core levels and valences and the standard deviation for each W-*n*O family presented as colored rectangles. The choice of standard deviation to describe the variability of the data set with respect to other indexes of dispersion is not casual. The standard deviation is affected by extreme outliers, while other indexes, such as interquartile range or average absolute deviation, are less or not at all affected by outliers and thus do not represent properly the entire data set. Anyway, to prove that our conclusions are not dependent on the index-specific choice, for comparison, we prepared the same graph using other dispersion indexes (see Figure 5S in the Supporting Information).

**Figure 4:**
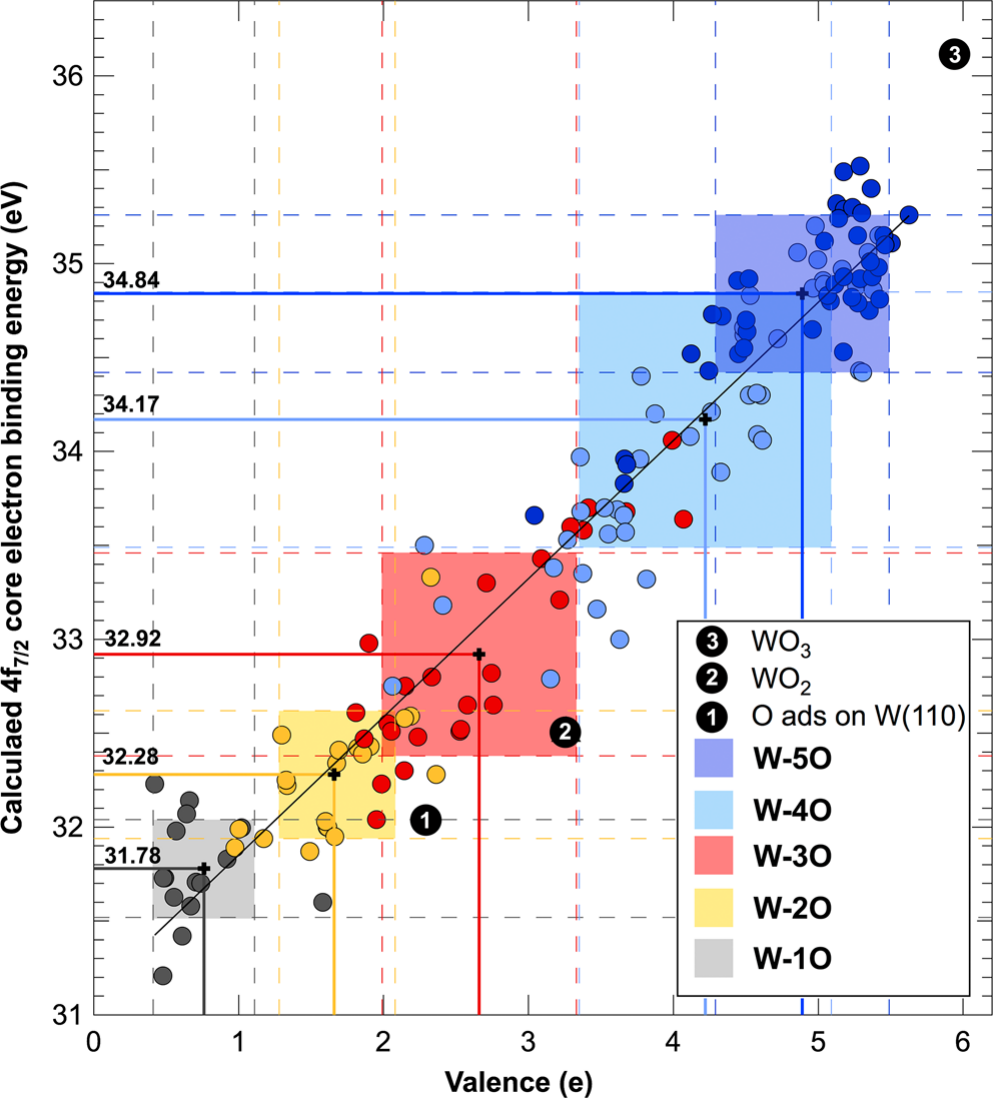
Dependence of the calculated W 4f_7/2_ core levels on the atomic valence. Each rectangle represents the standard deviation around the average values of the core levels and valence variables. Black lines stand for the data fit. For comparison, core-level values of W bulk and surfaces are also reported.

The data indicate that each distribution is well-centered on the linear fit line. The graph highlights two key effects: (i) for each different atomic coordination, the dispersion of core levels is substantial, ranging from 0.24 eV to over 0.68 eV, particularly for the 4O coordination, and (ii) in the specific cases of 2O–3O and 4O–5O atomic coordinations, we observe a remarkable overlap of the colored areas corresponding to different W-*n*O families. This result clearly demonstrates that an experimental measurement of core levels for small clusters, even if realized with high experimental resolution, does not allow for precise and direct determination of valency and, consequently, the atomic oxidation state. Notably, when plotting the interquartile range instead of the standard deviation, the overlap between different valence states becomes even more pronounced, with the 5O family that is almost incorporated into the 4O one.

The key for explaining our results lies in the high variability of interatomic distances when oxides are composed of only a few atoms whose arrangement is still quite different from that of the crystal structures formed when atomic aggregates are larger. In bulk tungsten, although the coordination with oxygen is the same, the average W–O bond length is fixed and different for each compound; when dealing with nanostructures, this statement does not apply, and the assignment of an oxidation state should be reviewed. As illustrated in Figure 3, W–O bond distances within the same coordination number exhibit considerable variability, which ranges as high as 0.3 Å for the W–3O and W–4O groups. This variability, stemming from the reduced crystallinity at the subnanometer scale, can significantly impact local chemical properties. The W–O bond length is indeed crucial in various tungsten-based catalysts. In Bi_2_WO_6_, for example, the presence of bismuth reduces the range of W–O bond lengths by 75% compared to WO_3_, leading to a redistribution of charge on W atoms that alters their valence state. As a result, W–O bonds in Bi_2_WO_6_ are more ionic, and this characteristic lowers the sensitivity and selectivity of this compound for NO_2_ with respect to WO_3_.^72^

The fact that the variability of local configurations does not allow the oxidation state of nanomaterials to be known directly and accurately using XPS has important consequences for the determination of the chemical properties of the catalysts. It is well-known that the valence state is crucial for a large variety of metal-oxide systems where reactivity, selectivity, and reaction patterns of catalysts are strongly influenced by it. This is the case, for example, for Pd(111) and PdO(101) surfaces, with the latter showing better performances for hydrogen oxidation to H_2_O_2_.^73^ Notably, the importance of the oxidation state increases even more at the nanoscale. The oxidation state of osmium single-atom catalysts (SACs), given by different coordination environment, is responsible for large hydrogen evolution reaction activity.^74^ The oxophilicity of metal atoms influences the oxidation state of single atoms during CO oxidation reaction, leading to different reaction orders and different rate controlling steps for Fe and Pd SACs,^75^ while different oxidation states in cobalt nanoparticles resulted in quite different temperatures of hydrogenation of CO and CO_2_.^76^

Here, we would like to emphasize that the heterogeneity of interatomic distances and valences, which both contribute to the determination of the oxidation state, is not a unique characteristic of tungsten oxide we studied; rather, it is expected across many nanocluster oxides. For this reason, we anticipate that the challenges in determining atomic oxidation states may extend to other oxides with little nuclearity (*N* < 30 ÷ 40) for which low crystallinity levels have been reported. This is the case of (TiO_2_)*_N_*, which shows noncrystalline properties only up to sizes of approximately *N* = 84,^77^ or in the case of ceria (*N* = 50).^78^ The large dispersion of structural variables characterizing nanoclusters has also been reported by Mammen et al.^79^ who found a large variability of Cu atoms Bader charge in Cu*_N_* (*N* = 4, 10, 20) nanoclusters caused by the inequalities of atoms and different local bonding environment. At the same time, the distribution of configurations in subnanometric catalysts can also represent a great opportunity because it allows us to obtain, on the same metal-oxide, local configurations in which binding energies of reactants, intermediates, and products are different, thus going beyond the growing interest of bifunctional catalysts. This result suggests the need to transcend the traditional concept of the single-active site when dealing with nanoclusters and push toward a new approach based on the statistical interpretation of the relationship between the structure of a nanocatalyst and its performance.

## Conclusions

In conclusion, we have demonstrated that high-energy-resolution X-ray photoelectron spectroscopy data do not facilitate rigorous and unambiguous assignments of oxidation states at the nanoscale solely based on comparison with bulk matter results. In this respect, a close comparison with the results of theoretical calculations is not only suggested but strongly recommended, although the solution to the problem is not straightforward. While the approach based on understanding properties at a local level is still extremely relevant, an in-depth understanding of the properties of low-nuclearity catalysts nevertheless requires the development of a statistical analysis that considers the intrinsic nature of the systems at the subnanoscale: the enormous degree of heterogeneity of the systems being studied.

## Experimental Methods

The Ru(0001) crystal was cleaned by cycles of Ar^+^ sputtering and annealing up to 1500 K in an O_2_ atmosphere. The residual oxygen has been finally removed by a flash annealing in the vacuum to 1500 K. Graphene was grown by chemical vapor deposition of ethylene (C_2_H_4_) at 1000 K in two stages: the first one with a pressure of 2 × 10^–8^ mbar, the second one with an increased pressure of 5 × 10^–8^ mbar to guarantee the complete covering of the surface.

W_13_^+^ and W_25_^+^ were generated by means of the laser ablation cluster source ENAC (Exact Number of Atoms in Each Cluster). The mass selection is performed by a quadrupole mass analyzer and is monitored by acquiring mass spectra (see Figure 1S in the Supporting Information). Size-selected tungsten nanoclusters were deposited directly in situ on graphene/Ru(0001), on which they are electrically neutralized, in soft-landing conditions, i.e., with a kinetic energy less than 1 eV/atom.^60^ The deposited cluster coverage was 0.06% ML (1 ML = 1.56 × 10^15^ atoms/cm^2^), and the amount of clusters deposited was monitored reading the cluster current on the sample. During depositions, oxidation and XPS measurements were performed and the temperature of the sample had been kept to 20 K to avoid clusters diffusion and nucleation, thus preserving the size selection.

High-resolution X-ray photoelectron spectroscopy (HR-XPS) measurements were performed at the SuperESCA beamline at the Elettra synchrotron facility (Trieste, Italy). Photoemission spectra were collected by means of a Phoibos 150 mm mean-radius hemispherical electron energy analyzer (SPECS) equipped with a delay line detector developed in-house. The overall energy resolution better than 80 meV for the photon energies and acquisition parameters employed. The XPS spectra were acquired by tuning the photon energy for having a photoelectron kinetic energy of about 100 eV to enhance the surface sensitivity. For each spectrum, the photoemission intensity was normalized to the photon flux and the BE scale was aligned to the Fermi energy of the Ru substrate. XPS spectra were fitted using the convolution of a Doniach–Sunjic^80^ line shape with a Gaussian line shape to account for experimental, inhomogeneous, and phonon broadening. Line shape parameters obtained were based on the least-square fit. The background was assumed to be a polynomial of the seventh grade.

The background removal has been a complicated procedure since the background changes with oxygen exposure. We decided to use the following strategy. We acquired the spectral region of the W 4f core level without the cluster. That region was then modeled using a polynomial and used as a background for metallic W nanoclusters spectra. For oxidized clusters, the polynomial was multiplied for a constant to account for changes due to oxygen adsorption. As the oxygen exposure increases, a new component arises at a binding energy of 31.2 eV in the higher-energy region of the valence band. This new component is attributed to O 2s core level of physisorbed O_2_ on the surface and must be taken into account. The spectrum of this component has been simulated using the line shape parameters derived from the work of Puglia et al.^91^ that have been kept fixed during the fitting procedure. Thus, the background for oxidized clusters has been modeled using the sum of the polynomial and the O 2s component, whose intensity was free to vary. W 4f core levels were fitted using the coordination families found from the theoretical analysis. Each family is made of the sum of *m* fitting functions, where *m* is the number of W atoms belonging to that family. This approach was aimed at fitting the experimental data through a chi-square minimization process. Alongside the binding energies obtained from DFT calculations, we incorporated a Gaussian function to describe the dominant contribution of vibrational and inhomogeneous broadening as well as a Lorentzian contribution to account for the core-hole lifetime in the photoemission process. For all spectral sequences, the Lorentzian widths were fixed, following optimization, at 0.07 ± 0.02 eV (for the 4f_7/2_ component) and 0.10 ± 0.02 eV (for the 4f_5/2_ component) for W_13_ and at 0.10 ± 0.02 eV (for the 4f_7/2_ component) and 0.13 ± 0.03 eV (for the 4f_5/2_ component) for W_25_. The larger widths obtained for the 4f_5/2_ spin–orbit split components are consistent with Koster–Cronig transitions. The Gaussian widths were determined to be in the range of 0.9–2 eV for all oxide-related components, while significantly smaller values (0.4 ± 0.1 eV) were observed for the metallic components. The asymmetry parameter of the Doniach–Sunjic (DS) function we used to analyze the spectra,^64^ which accounts for the probability of electron–hole pairs excitation, was included only for the metallic components, with values of 0.10 and 0.16 for W_13_ and W_25_, respectively. These components dominate at the onset of the oxygen uptake process, whereas the asymmetry for all components related to the oxide forms was found to be zero. Under these conditions, the DS function assumes the line shape of a Voigt function, which is commonly adopted to analyze data for metal-oxide systems.

## Theoretical Methods

The calculations have been performed using density functional theory (DFT) as implemented in the VASP code.^81^ The systems were described with a slab of 4 layers of Ru in a 12 × 12 hexagonal supercell and a layer of 13 × 13 unit cell of graphene placed on top with an overall number of 914 atoms, excluding W nanoclusters. The W clusters were relaxed in the gas phase (see Figure 6S in Supporting Information) and then placed on the valley of the corrugated graphene layer, with its center on a fcc site. The bottom two layers of Ru were kept frozen at their bulk geometry, with a lattice parameter of 2.724 Å, and the rest of the system was fully relaxed using the rev-vdw-DF2 functional^82^ until the largest residual force was less than 0.015 eV/Å. We employed the projector augmented method (PAW^83^) using PBE potentials.^84^ The plane-wave cutoff was set to 400 eV, and the relaxations were performed by sampling the Brillouin zone using the Γ point only. Core-level binding energies were calculated as the energy difference between two simulations: a standard reference calculation and a second one in which a core electron is promoted to the valence shell. This is achieved by adding an extra valence electron and assigning a modified pseudopotential that reflects the presence of a core hole. In the PAW formalism implemented in VASP, this process is handled internally during the calculation, eliminating the need to manually generate a separate PAW potential for the atom with a core hole. In both cases, the total charge density is iteratively driven to self-consistency, allowing for valence electron relaxation around the core hole. Using this approach, typical agreement between experiment and theory is better than 50 meV, as reported for several systems.^63,85–90^

The W clusters were placed, as neutral species because of charge neutralization by graphene, in the valley of the corrugated graphene layer, where the stronger interaction with the Ru(0001) substrate is known to increase its stability. The strategy employed for incorporating oxygen into the clusters before global structural DFT relaxation involved three key steps: (1) random selection of W atoms, (2) positioning of a single oxygen atom at a distance of 2 Å (this value was chosen because W–O bond lengths in W oxides are in the range 1.7–2.2 Å), with randomly chosen azimuthal and polar orientations for the W–O bond, and (3) repeating the procedure for the desired number of oxygen atoms, while adhering to the constraint that the maximum number of O atoms bonded to W atoms must be less than 7 (as seen in bulk tungsten oxides). For each oxygen density, the procedure was repeated 10^3^ times, with the final configuration selected for structural relaxation via DFT being the one with the lowest average O–O distance, as oxygen is known to diffuse and group in bulk tungsten.

## Supplementary Material







## Notes

Mass spectrum of W nanoclusters; W–W average bond length distribution graph; core levels versus Bader charge graph for each atom of oxidized W nanoclusters; core levels versus coordination graph; core levels versus Valence with interquartile ranges graph; ground state geometries of W_13_ and W_25_ nanoclusters (PDF)
Results for W_13_ clusters (AVI)
Results for W_25_ clusters (AVI)
