# Supplementary material for: Limitations in Determining Oxidation States in Condensed Matter at the Subnanometric Scale
Source: J Am Chem Soc. 2025 Jun 13;147(25):21501–11. doi: 10.1021/jacs.5c02242 (PMC12203604; doi:10.1021/jacs.5c02242)
Supplement: Supplementary file 1 [file ja5c02242_si_001.pdf]

# Supporting Information: Limitations in Determining Oxidation States in Condensed Matter at the Sub-Nanometric Scale

Deborah Perco[1], Monica Pozzo[2,3], Andrea Berti[1], Federico Loi[1,4], Paolo Lacovig[5], Silvano Lizzit[5], Aras Kartouzian[6], Ulrich Heiz[6], Dario Alfè[7,8] and Alessandro Baraldi[1,5,*]

[1]Department of Physics, University of Trieste, via Valerio 2, 34127 Trieste, Italy.

[2]Faculty of Technological & Innovation Sciences, Universitas Mercatorum, Piazza Mattei 10, 00186 Rome, Italy

[3]Institute for Materials Discovery, UCL East, Marshgate Building, 7 Sidings Street, Stratford, London, E20 2AE, United Kingdom

[4]J. Heyrovsky Institute of Physical Chemistry, Dolejškova 2155/73, 182 23 Prague, Czech Republic.

[5]Elettra Sincrotrone Trieste, AREA Science Park, 34149 Trieste, Italy.

[6]Chemistry Department & Catalysis Research Center, Technical University of Munich, Lichtenbergstr. 4, Garching D-85748, Germany.

[7]Department of Earth Sciences and London Centre for Nanotechnology, University College London, Gower Street, London, WC1E 6BT, UK.

[8]Dipartimento di Fisica Ettore Pancini, Università di Napoli Federico II, Monte S. Angelo, 80126 Napoli, Italy.

*KEYWORDS: tungsten, clusters, XPS, oxidation, valence state, metal-oxides, graphene, DFT*

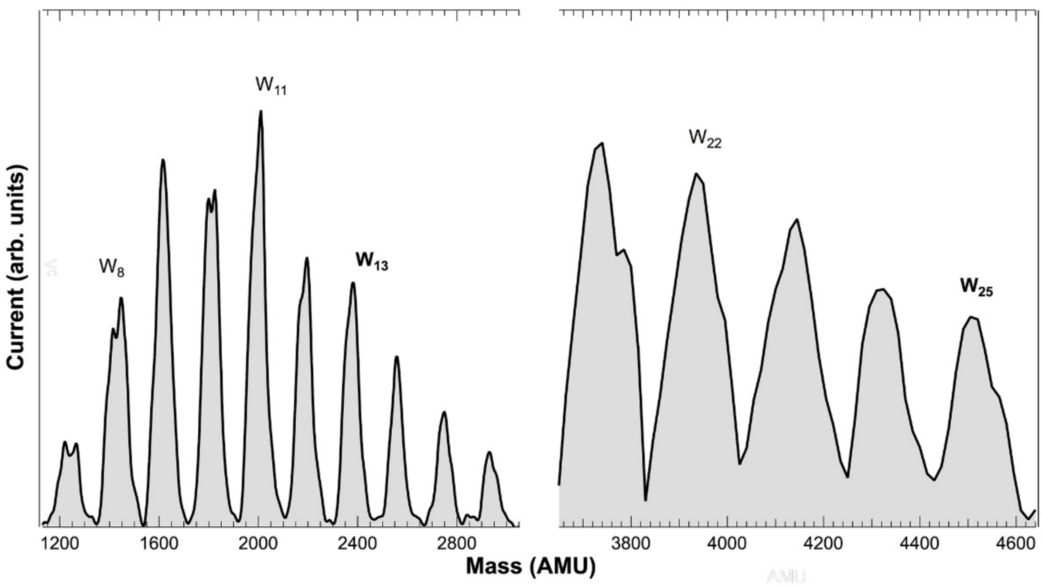


**Figure 1S.** The deposited clusters are highlighted in bold.

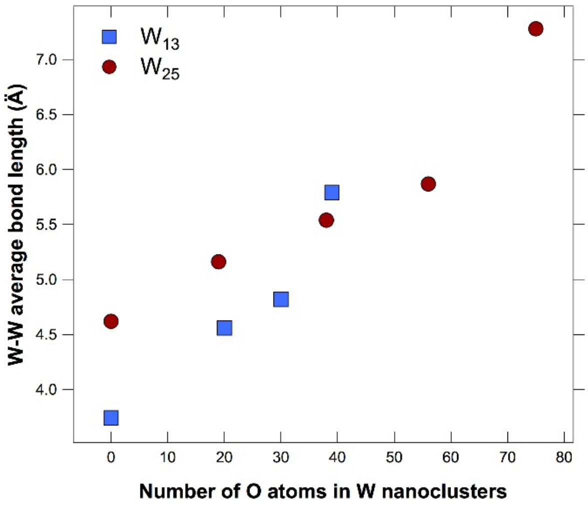


**Figure 2S.** W-W average bond length distribution as a function of O density in nanoclusters. Blue points represent $W_{13}O_x$ (x=0,20,30,40) while blue points represent $W_{25}O_x$ (x=0,19,38,56,75) nanoclusters.

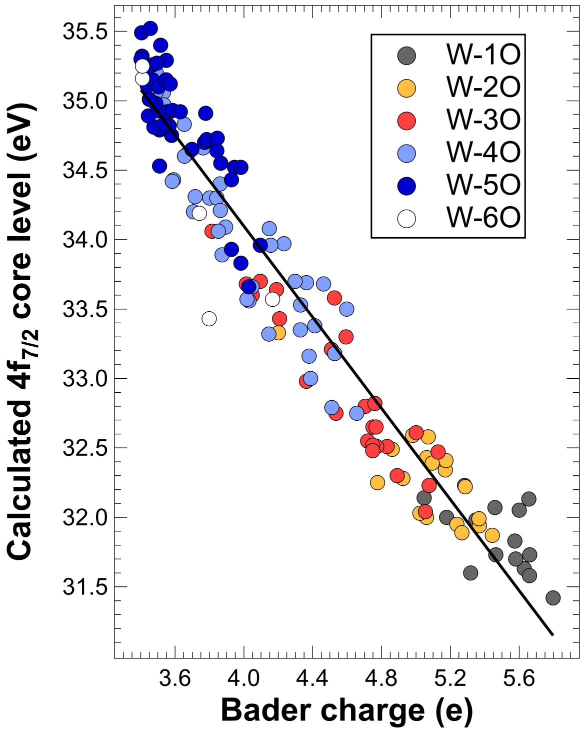


**Figure 3S.** Computed W $4f_{7/2}$ core levels of W atoms as a function of the Bader Charge of each atom. Different colours identify the different families.

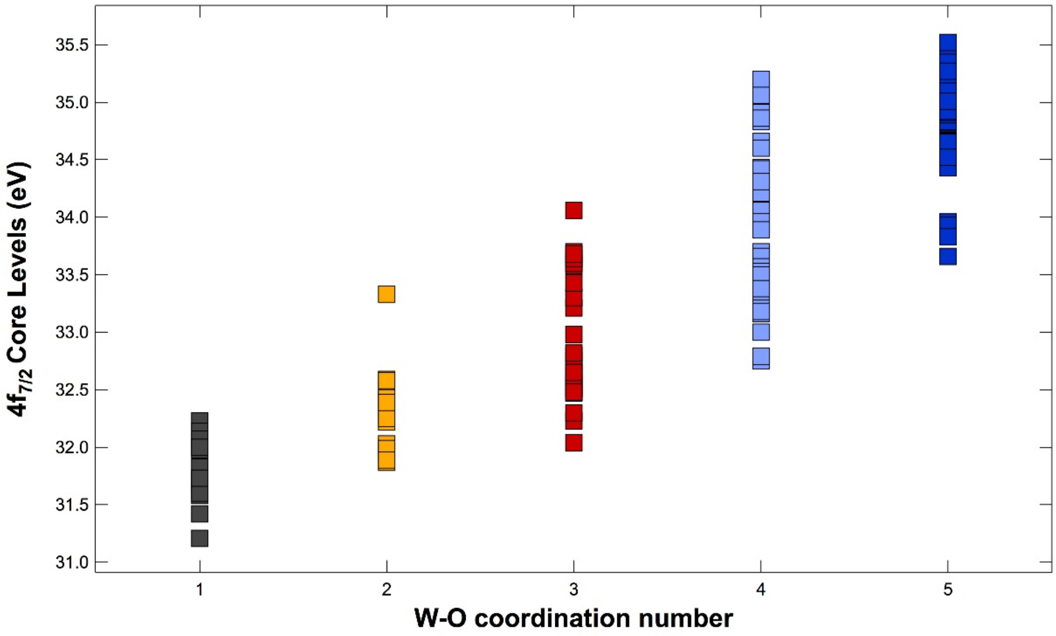


**Figure 4S.** Computed W $4f_{7/2}$ core levels of W atoms as a function of the coordination with O atoms. Different colours identify the different families.

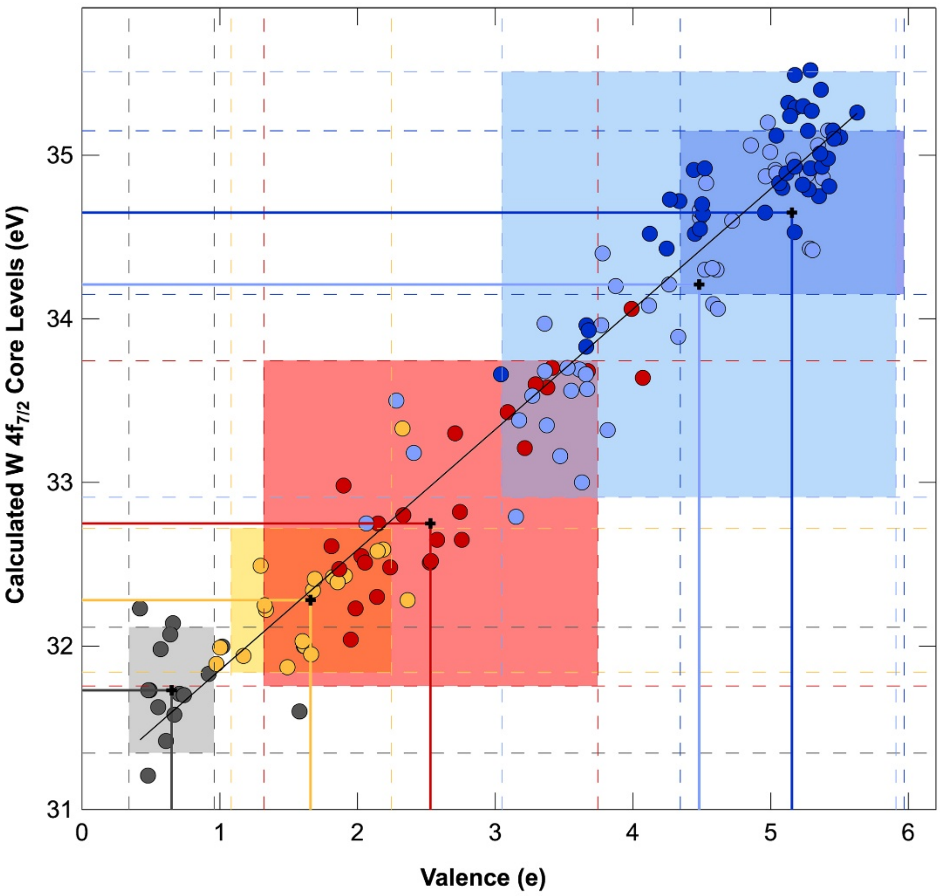


**Figure 5S.** Computed W $4f_{7/2}$ core levels of W atoms as a function of their valence. Each rectangle represents the interquartile range around the median values of core levels and valences. Different colours identify the different families.

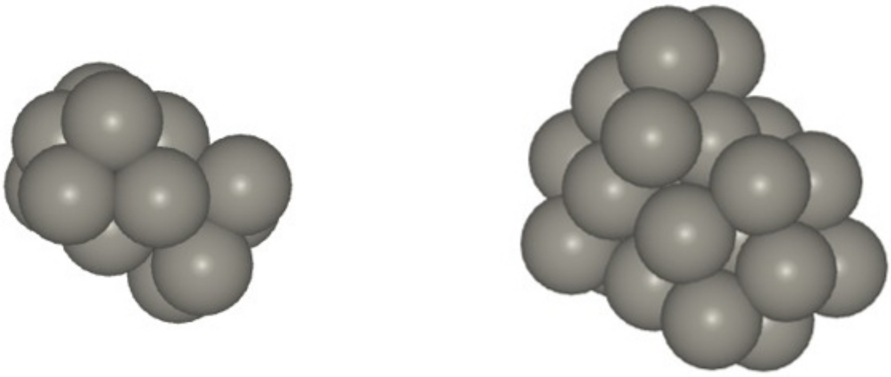

**Figure 6S.** Relaxed gas-phase geometries of $W_{13}$ and $W_{25}$ nanoclusters.
